# Label‐Free Leukemia Monitoring by Computer Vision

**DOI:** 10.1002/cyto.a.23987

**Published:** 2020-02-24

**Authors:** Minh Doan, Marian Case, Dino Masic, Holger Hennig, Claire McQuin, Juan Caicedo, Shantanu Singh, Allen Goodman, Olaf Wolkenhauer, Huw D. Summers, David Jamieson, Frederik W van Delft, Andrew Filby, Anne E. Carpenter, Paul Rees, Julie Irving

**Affiliations:** ^1^ Imaging Platform Broad Institute of MIT and Harvard Cambridge Massachusetts; ^2^ Northern Institute for Cancer Research Newcastle University UK; ^3^ Department of Systems Biology & Bioinformatics University of Rostock Rostock Germany; ^4^ College of Engineering, Swansea University, Bay Campus Swansea SA1 8EN UK; ^5^ Flow Cytometry Core Facility. Innovation, Methodology and Application Research Theme, Biosciences Institute, Newcastle University NE2 4HH UK

**Keywords:** machine learning, deep learning, computer vision, label‐free, leukemia, imaging flow cytometry, neural networks

## Abstract

Acute lymphoblastic leukemia (ALL) is the most common childhood cancer. While there are a number of well‐recognized prognostic biomarkers at diagnosis, the most powerful independent prognostic factor is the response of the leukemia to induction chemotherapy (Campana and Pui: Blood 129 (2017) 1913–1918). Given the potential for machine learning to improve precision medicine, we tested its capacity to monitor disease in children undergoing ALL treatment. Diagnostic and on‐treatment bone marrow samples were labeled with an ALL‐discriminating antibody combination and analyzed by imaging flow cytometry. Ignoring the fluorescent markers and using only features extracted from bright‐field and dark‐field cell images, a deep learning model was able to identify ALL cells at an accuracy of >88%. This antibody‐free, single cell method is cheap, quick, and could be adapted to a simple, laser‐free cytometer to allow automated, point‐of‐care testing to detect slow early responders. Adaptation to other types of leukemia is feasible, which would revolutionize residual disease monitoring. © 2020 The Authors. *Cytometry Part A* published by Wiley Periodicals, Inc. on behalf of International Society for Advancement of Cytometry.

## Graphical abstract

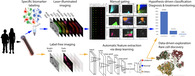

Label‐free deep learning analysis pipeline for acute lymphoblastic leukemia (ALL). A conventional, (blue path) flow cytometric approach to identify ALL cells uses multiple antibody markers and laborious, manual gating to achieve simple, low‐content reports. The approach proposed in this study (yellow path) involves no labels, using only bright‐field and dark‐field information. Deep neural networks might yield the needed information for diagnosis and treatment monitoring.

Quantification of persisting leukemia in bone marrow and/or peripheral blood during initial therapy has become standard of care to enable risk‐directed therapy, where more intensive therapy is given to slow responders, who are at a higher risk of relapse 1. Such stratification has contributed to current overall survival rates approaching 90% 2. In addition, it has allowed the de‐escalation of chemotherapeutic dose and thus lessened toxicity in children deemed to be at low risk of relapse, without impacting on cure 3.

Traditionally, persisting leukemia is assessed by morphology to define complete remission (<5% ALL cells), and to identify slow early responders, often defined as >25% ALL cells at Day 8 or 15 following therapy initiation. More sensitive techniques are then used to identify leukemia that is below the limit of detection based on visual assessment under a microscope, known as minimal residual disease (MRD). Malignant leukemia cells can be difficult for experts to accurately identify by morphology and can be mistaken for benign immature B‐cell precursors, particularly in regenerating marrow. Two recent studies have highlighted the need for more accurate methodologies to improve, and possibly replace, visual assessment of morphology in evaluating disease response 4, 5.

The two principal residual disease assessment methodologies for ALL are molecular analyses of antigen receptor gene rearrangements or flow cytometry of aberrant immunophenotypes 6. Fundamental to residual disease detection by flow cytometry is the characterization of a leukemia‐associated immunophenotype at diagnosis. This is a four to ten‐antibody assay, in which the leukemic cells fall into so‐called empty spaces within scatter plots, distinct from regions housing normal lymphocyte progenitors (Fig. S1 and S2). It can thus discriminate and quantify leukemic cells in “on‐treatment” samples 7. Both methods of ALL detection are highly specialized, require specific reagents and extensive training, and are thus slow, labor intensive, and costly. For resource‐poor countries, costs, travel, and access to specialist laboratories prohibit residual disease‐defined optimized treatment; and in some centers, even morphological analyses to determine complete remission or slow early response are done by tele‐pathology 8. In addition, leukemia cells in some children cannot be quantified by either method due to the absence of “trackable” molecular or cellular features for their particular leukemia 6.

**Figure 1:**
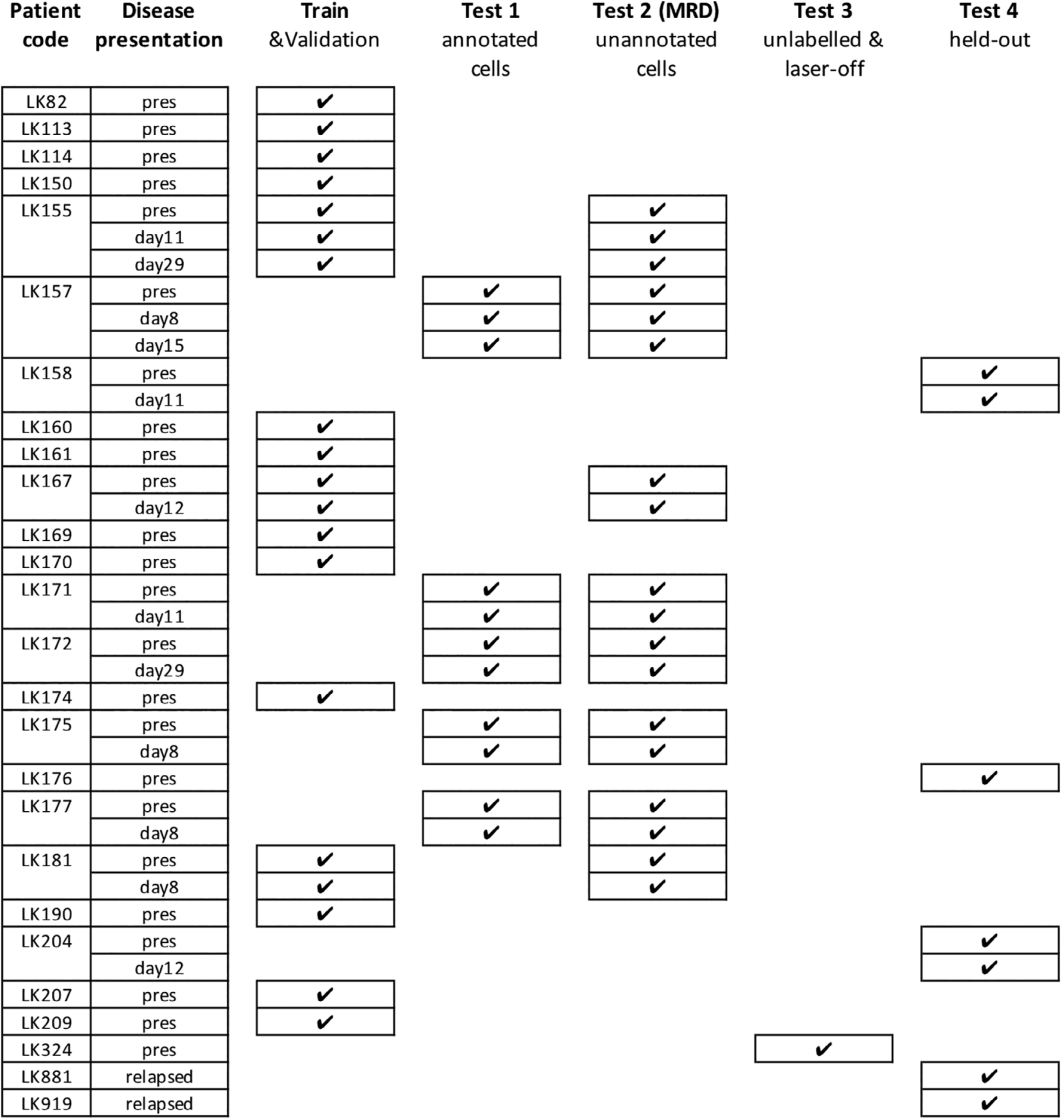
Sample partitioning strategy for training, validation, inference, and avoiding overfitting. Samples were split for training (including validation), testing (*Test 1–3*) and hold‐out (*Test 4*). Training/validation set contained pooled data of 19 entries from 15 patients. Samples were collected and measured at the time of presentation (abbreviated as “pres”) and after round(s) of treatments (noted as days after treatment). Test set 1 contained manually gated ground‐truth populations for leukemic blasts, normal lymphocytes and other cell types (Fig. 2A‐C). Test set 2, which contains DAPI‐positive, in‐focus single white blood cells, was designed to validate whether the learned algorithms were able to derive a correct residual disease (MRD) readout, that is, percentage of leukemic cells within the total number of white cells in the bone marrow sample (Fig. 2D). Note: Although some training data and Test set 2 were generated from the same patients, the training sets use a small number of individually annotated healthy/leukemic cells, while Test set 2 presents a large number of unannotated cells. Test set 3 (>200,000 single cells in total) was conducted with stained/unstained samples in a condition with or without laser illumination, confirming that the performance of the trained neural network was not dependent on the presence of bleed‐through fluorescence or lasers (Fig. 2E). Test set 4 was kept held‐out and only unlocked immediately before submission of the manuscript for the final verification of the success of the machine learning models (Fig. 2F).

Recently, flow cytometry has been integrated with fluorescence microscopy to create imaging flow cytometry (IFC), where an image of each cell is captured as it flows past a light source and a charge‐coupled device (CCD) detector 9. IFC combines the high throughput capacity of flow cytometry with the spatial image information of multiple fluorescence channels as well as the bright‐field, similar to the transmitted light image, and dark‐field, equivalent to side scatter in conventional flow cytometry 10. The high‐content data rapidly captured using IFC is well‐suited to classification of cell phenotypes by machine learning, particularly deep learning, given the large number of training images required to apply deep convolutional neural networks (Figs. [Link], [Link]). In previous work, we demonstrated the use of both traditional machine learning 11 and deep convolutional neural networks 12 to classify cell cycle phases in ~34,000 Jurkat cells using only the bright‐field and dark‐field channels from IFC, eliminating the need for cell cycle markers or DNA stains. We wondered whether deep convolutional neural networks might be able to detect leukemic cells from bone marrow samples of ALL patients using few or no fluorescent markers.

## Materials and Methods

### Patients

Bone marrow samples from children (less than 18 years) who presented with B lineage ALL within the northern region of England (October 2013 to July 2015) were used in this study. They were obtained from the Newcastle Hematology Biobank following project approval (reference 2002/11 and 07/H906). The children were registered on the UKALL2011 protocol and were treated with three to four drugs during the induction phase of treatment when on‐treatment samples were assessed. Clinical details of the patients used in the study are shown in Supporting Information Table S1.

### Flow Cytometry

Flow MRD was performed according to the UK standardized method 13. Briefly, samples were collected into Acid Citrate Dextrose and with red cells lysed using a standard ammonium chloride procedure. Biomarker labeling was done using fluorescent‐conjugated antibodies such as CD45‐APCH7, CD10‐PECy7, CD19‐APC, CD34‐PerCP (all from BD Biosciences, Plymouth, UK) and nuclear staining by Syto41 (Molecular probes, Loughborough, UK). Flow cytometric measurement was done on a BD FACs Canto II equipped with 488‐nm (blue), 633‐nm (red), and 405‐nm (violet) lasers, with a target number of 50,000 cells for diagnostic samples and >300,000 for “on‐treatment” samples. For analysis, an expert‐guided sequential manual gating strategy was applied: Lymphoid cells were first gated on forward and side scatters, followed by CD19^+^, low side scatter cells, then refined inspection on CD19^+^CD34^—^ and CD19^+^/CD34^+^ double positive cells. Finally, the expression of CD10 along with CD45 was assessed in the CD19^+^CD34^+^ and CD34^—^CD19^+^ populations to discriminate ALL cells from normal B cells. Samples were considered positive if the number of leukemic cells identified was equal to or greater than 0.01% (i.e. the percentage of leukemic cells over the total number of nucleated cells), providing at least 50 clustered events were apparent.

### Imaging Flow Cytometry and Ground Truth Generation

Bone marrow samples were lysed with Lyse/Fix 5x Buffer (BD Biosciences) and labeled with CD45‐APCH7, CD10‐PE, CD34‐PE Texas Red, CD19‐APC, DAPI, and p65‐FITC. The latter was included as an antigen not recognized to play a part in the leukemia‐associated immunophenotype (LAIP). IFC measurement was conducted using a dual camera ImageStream X MarkII system 10 (Amnis, Seattle, WA), equipped with 488 nm, 405 nm, 561 nm, and 642 nm excitation lasers. Data were collected at 40× magnification, pixel size 0.3 × 0.3 μm. Bright‐field illumination was collected in channels 1 (camera 1, 430 nm–480 nm) and 9 (camera 2, 570 nm–595 nm). Dark‐field illumination was collected in channel 6 (camera 1, 745 nm – 800 nm) from a 758 nm laser source. Emission from CD10‐PE was measured from the 488 nm laser in channel 3 (560 nm–595 nm), Texas Red emission was measured from the 488 nm laser in channel 4 (595 nm–660 nm), CD19‐APC from the 642 nm laser in channel 11 (660 nm–745 nm), CD45‐APCH7 from the 654 nm laser in channel 12 (740 nm–800 nm) and finally DAPI emission was measured from the 405 nm laser in channel 7 (430 nm–505 nm). Standard flow cytometric compensation procedure was applied in each sample.

Data analysis first started with the exclusion of clumped cells and out‐of‐focus cells based on aspect ratios, size, and gradient root mean square of typical noncellular events (Figs. S1 and S2). We then constructed pairwise 2‐D scatter plots and performed the manual sequential gating to identify ALL cells as for the standardized flow method. In addition, we identified normal B cells (CD19^+^, CD34^−^, CD45^+^, and CD10^+^/^−^) and classified cells with high side scatter, CD19^+^ and DAPI^+^ as “other”; while DAPI negative events were classified as red cell/debris. Gated cell populations were exported into a file container (.CIF) and served as the ground truth for downstream analyses. Bleedthrough (spillover) was examined in a separate experiment with labeled and unlabeled samples in laser‐on and laser‐off conditions (Fig. S6).

### Image Analysis

Images contained within a .CIF file were stitched into montages by using a Python script. Cellular objects from the montages were identified (segmented) using CellProfiler 3.1.6 14, 15. In the classical image analysis pipeline, object features were extracted by a series of built‐in measurement modules, including measuring object intensity, size, shapes, textures, correlations, and subcellular components. Data cleaning and feature selection were performed by Cytominer (https://github.com/cytomining/cytominer/) to remove features with near‐zero variance and features that have poor correlation across replicates. Redundant features that are highly correlated were then identified and only one feature for each of these groups was retained. After pruning, no pair of features had a correlation greater than the 95% cutoff threshold (Fig. S3, blue path).

### Classical Machine Learning

Various machine learning algorithms were tested and their hyperparameters were optimized by hyperopt 16 (https://github.com/hyperopt/hyperopt), including naive Bayes, random forest and support vector machine (SVM). We eventually chose linear SVM as the algorithm of choice for classical machine learning to achieve an acceptable balance between performance and computational efficiency. We trained the classifier to differentiate ALL cells from normal B lymphocytes with different combinations of antibody and DNA biomarkers. In parallel, we iterated the training–testing sets on 20 data sets (leave‐one‐instance‐out) to observe the variance of prediction accuracy due to the clinical diversity of patients.

### Deep Learning

Single‐cell images were exported from .CIF files. Data from fluorescent, bright‐field, and dark‐field channels were exported. The images were resized to 48 × 48 pixels by cropping the peripheral background or padding channel‐wise with noise sampled from the background of actual images (see code for details: https://github.com/carpenterlab/2019_doan_leukemia_submitted/blob/cb642b79fc6ee2c2ae26739147445e90c85ebbf1/deepometry/parse.py#L163). Additionally, cell images were contrast‐stretched channel‐wise to rescale the intensities between the 0.5 and 99.5 percentiles to the full range of uint8, [0, 256). We adopted ResNet architecture using a Python framework 17 (https://github.com/broadinstitute/keras-resnet). The network includes 50 convolutional layers, forming repetitive blocks that perform residual learning, followed by fully connected and softmax layers (Fig. S4). With larger ResNet architectures (e.g., ResNet200 18), we observed no improvements in accuracy or loss while there was an increase in training time and resources proportional to the increase in architecture size. Smaller models, such as a VGG‐like architecture with eight convolutional layers 19, performed well and were efficient on CPU, but a gap between training and validation accuracies indicated the opportunities to learn more features.

We computed categorical cross‐entropy as the loss function and accuracy as our metric, respectively. The model was compiled using the Adam optimizer with a learning rate of 0.0001. The learning rate was reduced by a factor of 10 when the validation loss failed to improve for 10 consecutive epochs. Training was set to stop after 25 consecutive epochs of no improvement in the validation loss. Objects were categorized as “leukemic,” “normal” (not leukemic), and “other” (nonlymphoid nucleated cells such as granulocytes, monocytes, dead or deformed cells). Training and validation data were randomly undersampled per‐patient across cell type to create a balanced data set. Eighty percent of sampled data was assigned to the training data set, with the remaining 20% assigned to validation.

The data were zero‐centered using channel‐wise mean subtraction. Means were precomputed from the training set. Mean subtraction and augmentation were performed in real time during training and validating operations. Augmentation included random combinations of horizontal or vertical flips, horizontal or vertical shifts (up to 50% of the image size), and rotations up to 180°. Augmented training and validation data were generated in batches of 256 images to maximize GPU memory resources. We configured the model to train for a maximum of 512 epochs, though early stopping generally terminated training before 200 epochs. Each epoch ran M/256 steps, with M as the number of training samples, to ensure the entire training set was seen once per epoch. Validation occurred once at the end of each epoch, using the entire validation set with validation step K/256, where K is the number of validation samples. Test data were comprised entirely of withheld patient data. Before prediction or evaluation, the mean pixel values obtained from the training data sets were subtracted from the test data. No other processing or augmentation was applied.

### Data‐driven exploration

Extracted features from the high‐content analysis pipeline or deep learning were converted into embeddings, which were then projected in Tensorboard embeddings visualization (URL2). For the classical high‐content image analysis procedure, we directly calculated t‐Distributed Stochastic Neighbor Embedding (t‐SNE)^20^ and principal component analysis (PCA) components from cell features measured by CellProfiler. For deep learning, we used the pooled features prior to the last fully connected layer as a feature extractor (pool5 layer of ResNet50). We then applied this feature set to the test data to obtain 2048 deep learning embeddings for each object in the data set. We then utilized Tensorboard built‐in t‐SNE and PCA functions to visualize the embeddings in 3D scatterplots. Using interactive gates on t‐SNE/PCA plots of TensorBoard, one also has options to isolate objects‐of‐interest for further analysis.

## Results

Using diagnostic and follow‐up bone marrow aspirates taken during remission induction from children with B lineage ALL and with the leukemia‐associated immunophenotype (*n* = 30, collected over two and a half years), we first trained a convolutional neural network to separate IFC cell images into three classes: ALL blasts (CD19^+^CD10^+^CD34^+^/^−^ and CD45^+^/^−^), normal B lymphocytes (CD19^+^CD10^−^CD34^−^ and CD45^+^), and “other” nucleated cells—denoting granulocytes, monocytes, deformed/dead cells etc.). We adopted ResNet architecture 17, which includes 50 convolutional layers, forming repetitive blocks that perform residual learning, followed by fully connected and softmax layers (see Methods, URL1, Supporting Information). Using all the image channels (fluorescently tagged antibodies, together with a nuclear dye and bright‐field and dark‐field) of the red‐cell‐lysed samples, this network predicted leukemic cells in unseen test samples with 98.2% accuracy, compared to a reference of gating on CD45/CD10/CD34/CD19 antibody stains following the United Kingdom‐standardized flow residual disease estimation method 13. (*Test 1*: Fig. 1, second column). We then sought to determine which biomarkers were essential by excluding them one at a time (during training and testing) and reassessing the classification performance.

**Figure 2:**
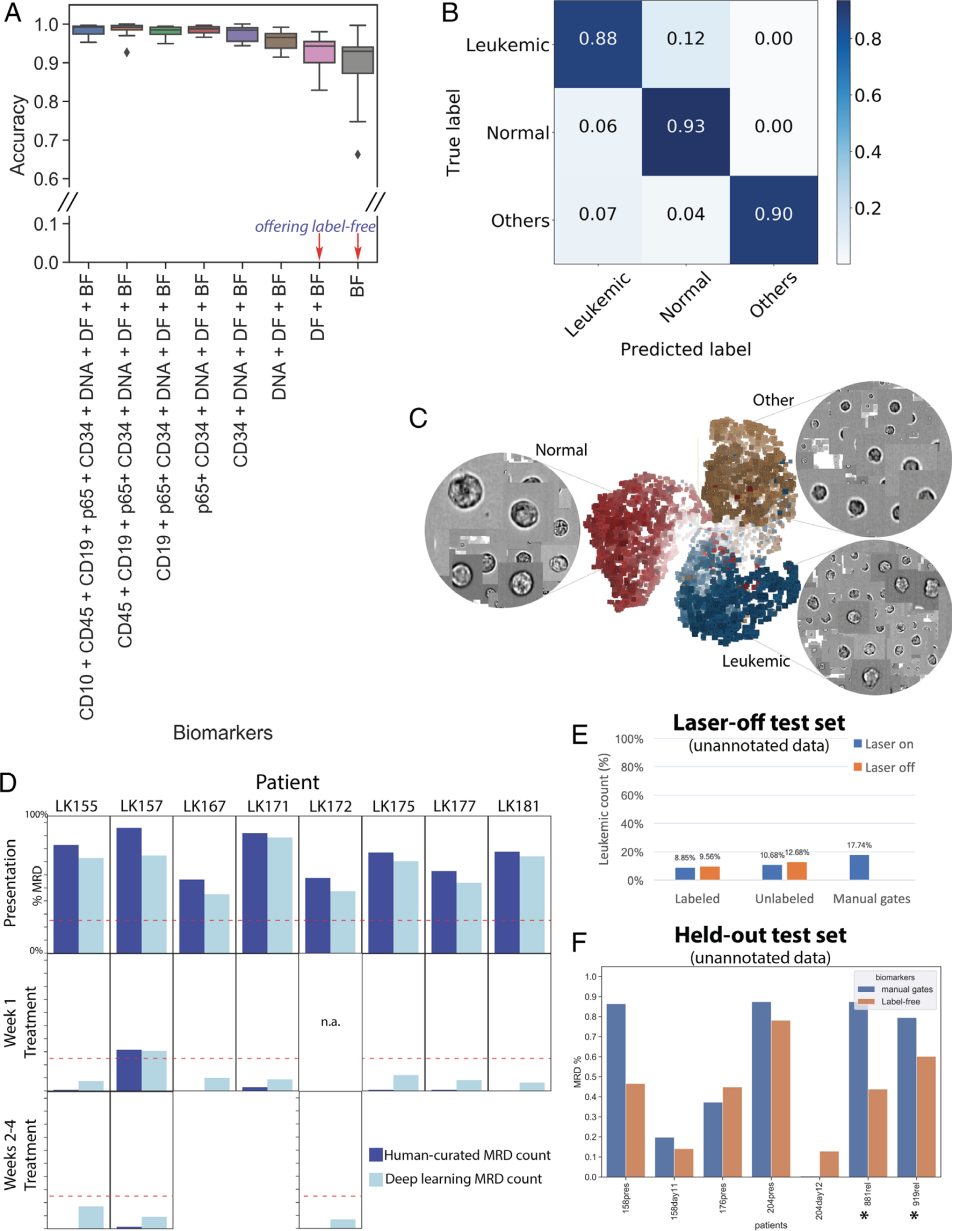
Label‐free identification of ALL cells by ResNet50 corresponds well with biomarker‐based analysis. **(A**) Prediction results on each of 11 test data entries from five patients, who have data at the time of presentation and during treatment (average accuracy from single‐cell classification) (*Test 1*). The first column reports average accuracy across samples using all channels; subsequent columns correspond to incremental dropping of the next channel. BF, bright‐field; DF, dark‐field. Boxplots show the median line, first and third quartiles. Whiskers are drawn to double interquartile range (+2IQR). Diamonds represent data events that are outside the low and high whisker ends. (**B**) Three‐class single‐cell classification using bright‐field and dark‐field channels (*Test 1*). Confusion matrix for three categories, n = 218,747 ground‐truth cells pooled from 11 test sets. (**C**) Clustering of 9,025 cells randomly sampled from patient LK157 at the time of presentation based on deep learning features. 3D t‐Distributed Stochastic Neighbor Embedding (t‐SNE)^20^ presentations based on 2,048 label‐free feature vectors for each cell from the second‐to‐last layer of the trained neural network, ResNet50, are shown. The t‐SNE calculation was stabilized after 400 iterations at perplexity of 30. Colors coded according to true class labels: Leukemic cells (blue), normal B lymphocytes (red), other cell types (yellow). The magnified images display true bright‐field channel, randomly zoomed at each cluster. Example data with overlaid images http://projector.tensorflow.org/?config=raw.githubusercontent.com/minh‐doan/Deeplearning_LabelFree_Leukemia/master/Publish/DL_supervised/Data/Step4/Output/projector_config.pbtxt can be visualized with a web‐based projector. (**D**) Comparison between human‐curated manual gates and label‐free deep learning in predicting residual disease fraction (*Test 2*). The prediction on each sample was performed on the DAPI‐positive population of in‐focus, white blood cells, which is a mixture of leukemic blasts, normal B lymphocytes, other mature and immature hematopoietic cells such as granulocytes and monocytes. Red dash‐line is 25%—the threshold of treatment effectiveness of chemotherapy. (**E**) Summary of residual disease estimated by deep learning using information in bright‐field channel of labeled/unlabeled samples (each with laser on/off settings) (*Test 3*). Residual disease fraction was calculated as the percentage of predicted leukemic cells over the whole population of unannotated in‐focus single cells. For reference, human‐curated manual residual disease fraction (last blue column) was estimated based on the stained version with laser on. (**F**) Result of label‐free deep learning residual disease readout (yellow columns) on unannotated in‐focus single cells in held‐out data (*Test 4*). Blue columns are residual disease readout reported by standard clinical flow cytometric protocol. LK881 and LK919 (asterisks) are relapsed ALL patients. [Color figure can be viewed at wileyonlinelibrary.com]

Surprisingly, removing several fluorescent biomarker channels did not dramatically reduce the ability of the network to detect leukemia cells. In fact, using only the label‐free images (bright‐field and dark‐field) achieved a rate of 90.3% in leave‐one‐out testing (Fig. 2A) and 88.6% on patient samples completely excluded from the training protocol (Fig. 2B). This finding aligns well with other recent developments in label‐free bioimage analysis using deep learning 21, 22, which reconstruct fluorescent label channels from bright‐field images only. These advances demonstrate that label‐free imaging contains more information than what is visible to the naked eye, and deep convolutional neural networks can accurately recover this hidden information. Consistent with the high accuracy of label‐free classification, the features extracted by the penultimate layer (Fig. S4) of our trained deep network suffice to position cells into clusters that corresponded well with the true class identities (Fig. 2C).

For comparison, conventional machine learning (linear SVM) on morphological features, extracted by standard image processing 14, 15, yielded accuracies ~4% to 15% lower to that of deep learning (Fig. S5). While improvements might be possible by further algorithm selection and tuning, deep learning offers the advantage of eliminating several steps in the traditional machine learning workflow—such as segmentation, feature extraction, and feature selection—which significantly simplifies the ALL detection protocol. In addition, the huge numbers of cells available from clinical samples are a good fit for further improving the deep learning model in the future. We also observed that the chosen ResNet50 architecture performed better on our single‐cell IFC images of size 48 × 48 pixels than very large networks (such as ResNet101, ResNet200) or small models such as VGG (see Methods and Supporting Information).

Next, we validated the strategy using clinically relevant metrics in a held‐out set of samples. We used the trained neural network to estimate the number of leukemic cells in unannotated white cells (in‐focus singlets) to compute the leukemia percentage for each patient (*Test 2* in Fig. 1 third column, Figure 2D). This readout is the standard diagnostic score used in clinical practice to determine patient status. We compared the leukemia burden obtained with neural network predictions against that reported in the patient's record to estimate the performance of our proposed method in the real‐world diagnostic task. The network identified leukemic cells from a mixed population of bone marrow cells with an accuracy of >88% based solely on the bright‐field and dark‐field images of IFC, comparable to the use of a full panel of four specific antigenic biomarkers and human‐curated ALL counts. The commonly accepted cutoff to define slow early responding patients is 25% MRD 2, 3, shown as a red dashed line in Figure 2D. At this cutoff, sensitivity and specificity of our method was 100%, that is, the method identified all true positives (>25% leukemia cells) and true negatives (<25%). At a 10% residual disease cutoff, sensitivity remained at 100% but specificity lowered to 80% (Fig. 2D; LK175 (Day 8) and LK155 (Day 28) are discordant, with the label‐free method overestimating residual disease).

To confirm that the trained machine learning algorithms were not affected by bleed‐through fluorescence or by the presence of laser illumination in the label‐free channels, we measured unlabeled samples with the instrument laser fully off (*Test 3* in Fig. 1, Figure 2E, and S6). A bone marrow sample after Day 8 of treatment initiation (containing >1 million cells) was split into two portions; one was stained with fluorescent labeling reagents and one was left unstained, each was further split into two parts, one was measured with laser excitation (laser‐on) and one with all lasers turned off. Whether samples had been labeled or left unlabeled, and whether the laser was on or off, leukemia burden was 9–13% (Fig. 2E). The final test set was kept held‐out and only unlocked immediately before submission of the manuscript for the final verification of the success of the machine learning models (Fig. 2F). Here, the results reaffirmed the performance of the neural network on label‐free images to resemble human manual evaluation using labeled data, achieving 100% sensitivity and specificity at the level of 25% leukemic load.

## Discussion

In summary, we demonstrate identification of residual leukemic cells in clinical samples without specific antibody tags. Although the use of multiple antibody‐conjugated markers provides 11 percentage points better in classification accuracy, we observed that the morphological features from label‐free channels were still sufficient to achieve clear discrimination of ALL cell phenotypes, even when they are a very small percentage of the total white cells. The strong performance on hold‐out patient samples indicates robustness to technical and patient heterogeneity. Clinical deployment would require a careful collection of data across sites and operators to train a robust deep learning model for each type of instrument and sample preparation protocol. Nevertheless, eliminating the use of laser‐based instruments and staining protocols should inherently reduce patient‐to‐patient and facility‐to‐facility variations. We provide open‐source scripts https://github.com/carpenterlab/2019_doan_leukemia_submitted to facilitate reproducibility of the study, testing on expanded samples, and application to new clinical and basic biology problems.

Although this study is to our knowledge the first to test deep learning on counting ALL cells label‐free using imaging flow cytometry, it is consistent with related strategies that took alternate sample measurement or different computational approaches on other blood disorders. For example, Matek et al. used convolutional neural networks to achieve human‐level recognition of blast cells in acute myeloid leukemia (AML), using expert microscopic examination of histological stained blood smears on glass slides instead of a flow‐based system 23. The approach was not label‐free, required smearing samples, and unlike our presented approach it relied on subjective inputs from experts rather than biomarker labeling for training. Ugele et al. used label‐free digital holographic microscopy to achieve convincing classification of nine leukocyte types, as well as different leukemia subtypes principally at diagnosis 24. This is unfortunately not suited to minimal residual disease detection because a high throughput modality is required (such as imaging flow cytometry)—large numbers of cells need to be measured to find the rare leukemic cells. As well, that study did not use convolutional neural networks but instead SVM trained on conventional features from the images, which is limited to preengineered features. Kobayashi et al. 25 demonstrated label‐free optofluidic time‐stretch microscopy, creating a SVM classifier to identify paclitaxel‐treated MCF‐7 cells versus untreated cells at an accuracy of 92%. Also on a microfluidic flow platform, Dannhauser et al. 26 used light scattering properties to discriminate peripheral blood mononuclear blood cell types, including T‐, B‐lymphocytes, and monocytes in different stages of lymphoid and myeloid leukemia. Lee et al. 27 used an ultrafast quantitative phase imaging (QPI) flow cytometer to classify multiple human leukemic cell types at ~92–97% accuracy based on subcellular biophysical profiles. Similarly, Mugnano et al. 28 used QPI to detect characteristic morphologies of red blood cells in several inherited anemias, such as iron‐deficiency anemia, thalassemia, hereditary spherocytosis, and congenital dyserythropoietic anemia. We believe our results will encourage others to combine the properties of many of these studies and develop new methods for translational disease monitoring using deep learning on label‐free samples from commercially available instrumentation.

If adapted to clinical use, label‐free approaches could offer simplicity and robustness, as well as time and cost savings. While the presented strategy does not have the accuracy to completely replace morphology or residual disease tests per se at this stage, the accuracy is acceptable for the clinically meaningful cutoff level of 25% ALL cells, which may be especially useful in resource‐poor regions, which lack trained hematopathologists. It may allow slow early responders to be easily detected and prioritize the borderline cases for assessment by experts. As well as assessing treatment response in ALL, it may also be feasibly applied to diagnose ALL and to differentiate ALL from lymphoblastic lymphoma, as a discriminatory parameter is the level of leukemic cells in the bone marrow.

Looking further to the future, the requirement of only bright‐field and dark‐field data could catalyze an opportunity for a simplified, label‐ and laser‐free, hand‐held imaging cytometer that would allow automated, point‐of‐care residual disease testing. A simple, compact, lightweight, optofluidic system already exists; it is fitted with a cheap, diode light source and attached to a cell phone 29. While our network took weeks to train on a GPU (NVIDIA Titan X), once the classifier is learned, millions of cells might be imaged by a simplified cytometer, examined by the trained algorithm, and a residual disease readout delivered within minutes (for cell images of size 48 × 48 pixels, the typical inference speed is ~375,000 cells/min on a Titan X at batch size of 512). We would expect the accuracy and robustness of our method to improve with the expansion of the training data set, a tactic that can close the accuracy gap between clinical specialist and machine learning 30.

Our results provide a concrete example of deep learning on single cells to enable personalized medicine. While the focus here is ALL, residual disease monitoring is a key response biomarker in all chronic and acute leukemias, multiple myeloma, and some nonhematological cancers for circulating tumor cells 31, 32, 33. We expect the strategy and code provided here to have broad clinical applicability across a range of cancer types.

## Code Availability

Image analysis script (CellProfiler pipeline), Data processing script (Python), Classical machine learning scripts (Python, R), Deep learning scripts (Python) and template for data visualization (IDEAS) are available at URL1.

## Data Availability

Example data (anonymized) are available upon reasonable request. Please send request to J.I. julie.irving@newcastle.ac.uk.

## Supporting information


**MIFlowCyt**: MIFlowCyt‐Compliant Items

**Figure S1** Fluorescent biomarkers for identifying leukemic cells by imaging flow cytometry. A: Example images of normal (mature B) lymphocytes. B: Example images of lymphoblastic leukemic cells. Pseudocolors in each gallery: grayscale: bright field, green: FITC‐p65, yellow: PE‐CD10, orange: PETexas Red‐CD34, magenta: dark field, solid red: DAPI, hollow red: APC‐CD19, pink: APCH7‐CD45. **C**: Manual sequential gating to quantify leukemic MRD according to standard flow cytometric quantification. The gated population of normal lymphocytes (CD19^+^CD10^—^CD34^—^ and CD45^+^), leukemic blasts (CD19^+^CD10^+^CD34^+^/^—^ and CD45^+^/^—^) and other nucleated cells were also exported as ground‐truth training datasets for machine learning algorithms.

**Figure S2** Conventional gating template for identifying leukemia and normal B lymphocyte cell subpopulations. The conventional (and up‐to‐date, reliable) assessment of leukemic disease state was done using manual sequential gating of dot plots. **A**: We used DIVA software on traditional standardized flow cytometric data (without images) to discriminate leukemic blasts, normal lymphocytes, and other cell types based on fluorescently labeled antibodies (as opposed to Fig. S1C showing imaging flow cytometry gates and software). B: Equivalent manual analysis was also performed on the fluorescence channels of imaging flow cytometry data, again based on fluorescently labeled antibodies. Extra preprocessing steps (top right scatterplots) were done to exclude out‐of‐focus cells and clustered cell clumps. The multi‐step pipeline was abstracted for clarity.

**Figure S3** Comparison between automated image analysis pipelines. **Blue path (upper)**: High‐content classical image analysis protocol, which includes image preprocessing, object detection, extraction of pre‐defined morphological features by using image analysis software CellProfiler, feature selection by Cytominer, and subsequent use for traditional machine learning algorithms. Because of the sequential nature, every tuning in the upstream part of the pipeline might require complete redo of the full pipeline. **Yellow path (lower)**: deep learning protocol, which directly takes raw images as inputs and delivers phenotypic classification as output. Using ResNet50 architecture, feature extraction, feature selection and learning optimization altogether are integrated in a single framework. The convolutional network operates with multiple levels of representation learning, in which the representation at one level (starting with the raw input images) is subsequently transformed into more abstract representations at higher levels. Higher layers of representation amplify features of the input image that are important for discrimination and suppress irrelevant variations. Classification of cells, MRD readout and data visualization are common outcomes for both protocols.

**Figure S4** Architecture of the neural network ResNet50 used in this study. The ResNet architecture was implemented using open‐source Tensorflow and Keras frameworks written in Python. The network includes 50 convolutional layers, forming repetitive blocks that perform residual learning, followed by fully connected and softmax layers. Bottom of left panel continues on to the top of the right panel.

**Figure S5** Performance of linear support vector machine (SVM) on predicting leukemic cells, based on pre‐defined features extracted by image analysis software CellProfiler. Data from 20 patients who were at their first presentation to the clinic or after 1–4 week(s) of chemotherapy. Two cross‐validation operations were conducted in parallel: leave‐one‐label‐out (horizontal axis) and leave‐one‐patient‐out, i.e. each individual patient was tested by the classifier that was trained by a pooled dataset of 19 other patients. Boxplots show the median line, first and third quartiles. Whiskers are drawn to double interquartile range (+2IQR). Diamonds represent data events that are outside the low and high whisker ends.

**Figure S6** Examination of the effects of fluorescent labeling reagents and laser excitations on the efficiency of MRD measurement by deep learning. A sample of a patient at Day 8 treatment was split into 2 portions; one was stained with fluorescent labeling reagents and one was left unstained, each was further split into two parts, one is measured with laser excitation (laser‐on) and one is with all lasers turned off. **A**: The fluorescently labeled sample examined in laser‐on mode. **B**: Unlabeled sample without laser excitation. Note that bright‐field signals (channels 1 and 9) were still visible without laser excitation and nevertheless well‐suited for deep learning algorithm.

**Table S1** Clinical details of patients used in the study.

**Appendix**S1: Supplementary materials
