# Supplementary material for: Label‐Free Leukemia Monitoring by Computer Vision
Source: Cytometry A. 2020 Feb 24;97(4):407–14. doi: 10.1002/cyto.a.23987 (PMC7213640; doi:10.1002/cyto.a.23987)
Supplement: Supplementary file 1 — MIFlowCyt: MIFlowCyt‐Compliant Items [file CYTO-97-407-s002.doc]

|  |  |
| --- | --- |
|  |  |
|  |  |
| **Requirement** | **Please Include Requested Information** |
| 1.1. Purpose | To identify and gate B-cell leukaemic cells in patient bone marrow (flow).  To visualise B-cell leukaemic cells independent of cell surface markers, using bright field and dark field signals and deep learning algorithms (image stream). |
| 1.2. Keywords | Immunology, Oncology, Haematology, Label-Free, Deep Learning, Machine Learning. |
| 1.3. Experiment variables | Cell surface marker expression and viability of leukaemic cells (flow).  Bright field (BF) and dark field (DF) values for label free image stream figures (image stream). |
| 1.4. Organization name and address | Newcastle University Wolfson Childhood Cancer Research Centre, Level 6, Herschel Building, Brewery Lane, Newcastle upon Tyne, NE1 7RU |
| 1.5. Primary contact name and email address | Prof. Julie Irving. julie.irving@newcastle.ac.uk |
| 1.6. Date or time period of experiment | Samples ran between August 2013 to July 2015 |
| 1.7. Conclusions | We report for the first time the use of artificial intelligence of single cell images as a clinical tool for MRD detection. This novel method is antibody free, using only the morphological features extracted from bright field and dak field cell images from an imaging cytometer. The method is cheap, quick, highly applicable and could be performed with a simple, laser-free cytometer to allow point of care testing.  When all parameters were considered, correct classification of MRD cells occurred at a rate of 98.21% but even without antibody-conjugated signals, relying only on DAPI, DF and BF signals, correct cell classification averaged 85.36%. Finally, using only label-free DF/BF parameters, our classifier gave correct leukaemic cell call rates of 81.62%, in both diagnostic samples and importantly in ‘on treatment’ bone marrow samples. |
| 1.8. Quality control measures | Flow Cytometry  Canto II system was calibrated prior to use using CS&T Research beads (BD Biosciences, USA).  Image Stream  The system was fully ASISST calibrated with additional quality control performed using Cyto Cal 6 peak beads (FC3MV, Thermo Fisher, USA).  Single stained controls were collected with bright field illumination and scatter laser turned off in order to generate a compensation matrix post-acquisition as described previously (Filby and Davies 2012). |
| 2.1.1.1. (2.1.2.1., 2.1.3.1.) Sample description | Bone marrow derived human B-cell leukaemia cells. |
| 2.1.1.2. Biological sample source description | Human bone marrow. |
| 2.1.1.3. Biological sample source organism description | Human. |
| 2.1.2.2. Environmental sample location | Fixed frozen bone marrow cells. |
| 2.3. Sample treatment description | Bone marrow samples were fixed using Lyse/Fix buffer (BD Biosciences, USA) and frozen using 10% DMSO 90% FBS freezing media and stored at -80C prior to use.  Training samples were labelled with cell surface fluorescent labels. |
| 2.4. Fluorescence reagent(s) description | Cell surface markers used; CD19 (APC), CD10 (PE), CD34 (Texas Red), CD45 (APC-H7), and DAPI (BD Biosciences, USA) (Flow Cytometry) |
| 3.1. Instrument manufacturer | BD Biosciences (Flow Cytometry) Luminex Corporation (Image Stream) |
| 3.2. Instrument model | FACS Canto II (Flow Cytometry) Amnis, Imagestream X Mark II (Image Stream) |
| 3.3. Instrument configuration and settings | Canto II  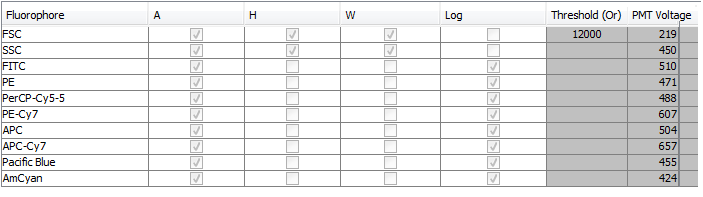  Image Stream Set up  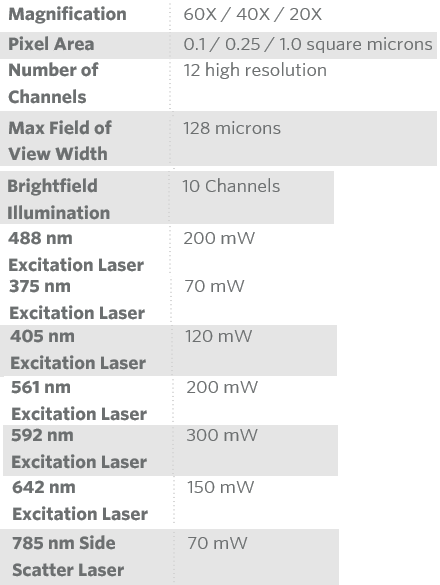 |
| 4.1. List-mode data files | *We recommend all authors to submit their data files to [http://flowrepository.org](http://flowrepository.org/) and to make them available for the peer-review process. If you have done so, please let us know by inserting the following codes (replace the red text):  1) The link for peer-review process:  http://flowrepository.org/id/RvFrxxxxxx (copy and paste the code). This link will only be shared with reviewers of your manuscript.  2) The repository identifier:  http://flowrepository.org/id/FR-FCM-xxxx (copy and paste the code). This link will be made publicly accessible after the paper is published. |

**Cytometry Part A**

**Author Checklist: MIFlowCyt-Compliant Items**

| 4.2. Compensation description | Canto II Compensation  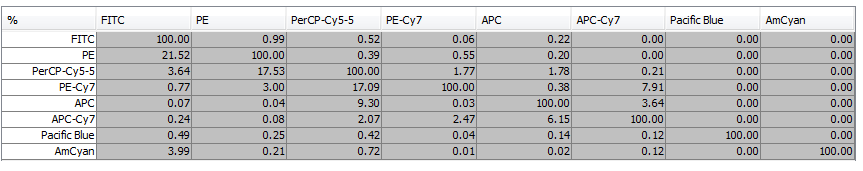  Image Stream Compensation  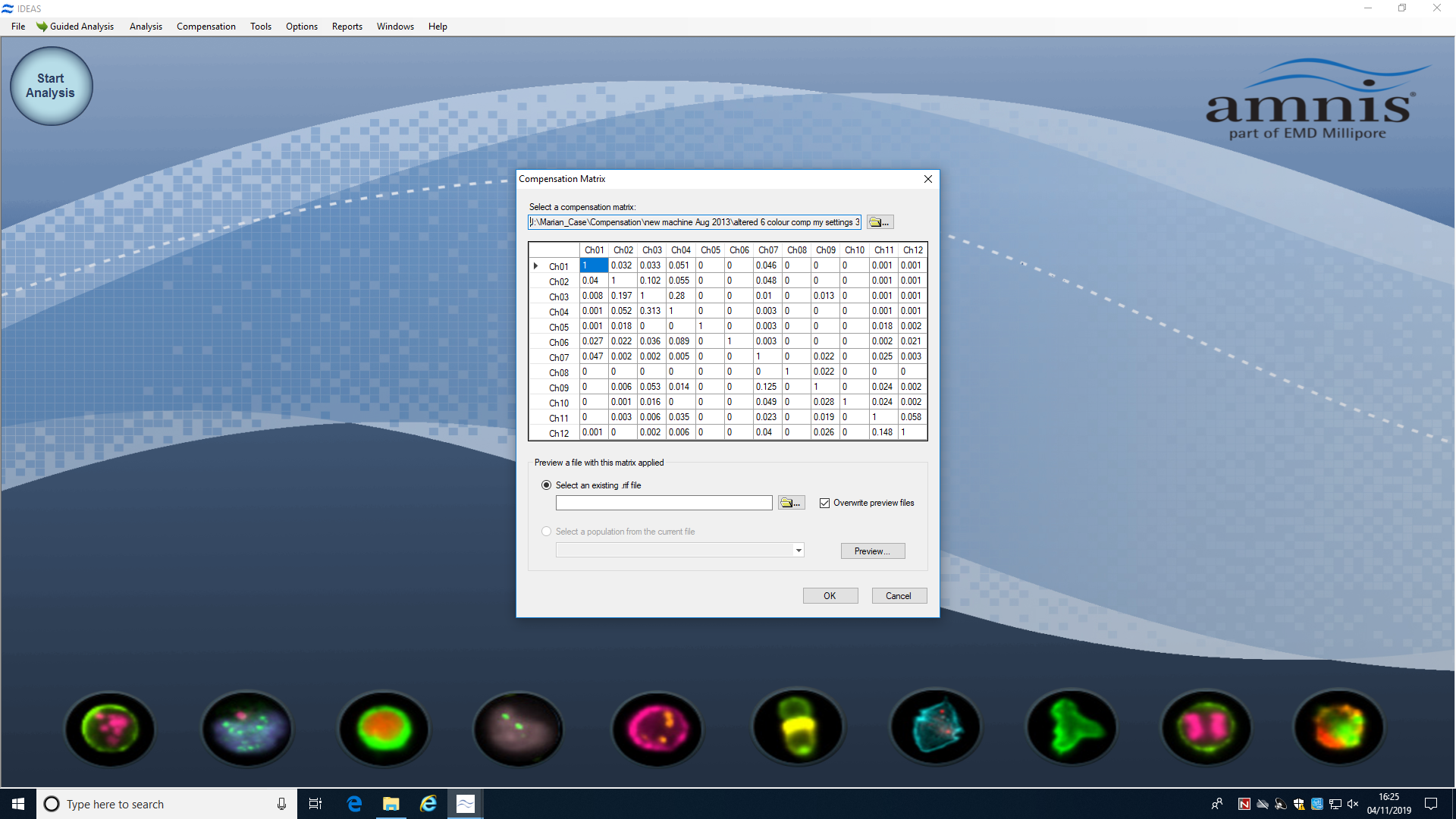 |
| --- | --- |
| 4.3. Data transformation details | Image Stream  Images contained within a .CIF file was stitched into montages by using a python script. Cellular objects from montage images were identified (segmentation) by using CellProfiler (Carpenter et al. 2006). Subsequently, object features were extracted by a series of built-in measurement modules, including measuring object intensity, size, shapes, textures, correlations, adjacency relationships between neighbor cells and subcellular components.  Data cleaning and feature selection was performed by Cytominer to remove near-zero variance, remove features that have poor correlation across replicates, remove redundant features that are highly correlated and retain only one feature for each of these groups. After pruning the feature tables, no pair of features have a correlation greater than 95% cut-off threshold. |
| 4.4.1. Gate description | Samples were gated on FSC vs SSC to remove debris, and combinations of cell surface markers were used to identify leukaemic blasts. (Flow Cytometry) |
| 4.4.2. Gate statistics | See table below |
| 4.4.3. Gate boundaries | NA |

Gating of analysed samples

**Notes**

Feel free to use more space than allocated.

You can embed graphics/figures in this document, if needed.

Please make sure to save the document in Microsoft Word version 2003 or older, before uploading to ScholarOne Manuscripts. When uploading this checklist to ScholarOne Manuscripts, please choose the “Supplementary Material for Review” category.

Please note that if your paper is accepted, the checklist will be published as an Online Supporting Information.

For any questions, please contact the Cytometry Part A editorial office at [Cytometrya@wiley.com](mailto:Cytometrya@wiley.com).
