# Supplementary figures and images for: Label‐Free Leukemia Monitoring by Computer Vision
Source: Cytometry A. 2020 Feb 24;97(4):407–14. doi: 10.1002/cyto.a.23987 (PMC7213640; doi:10.1002/cyto.a.23987)

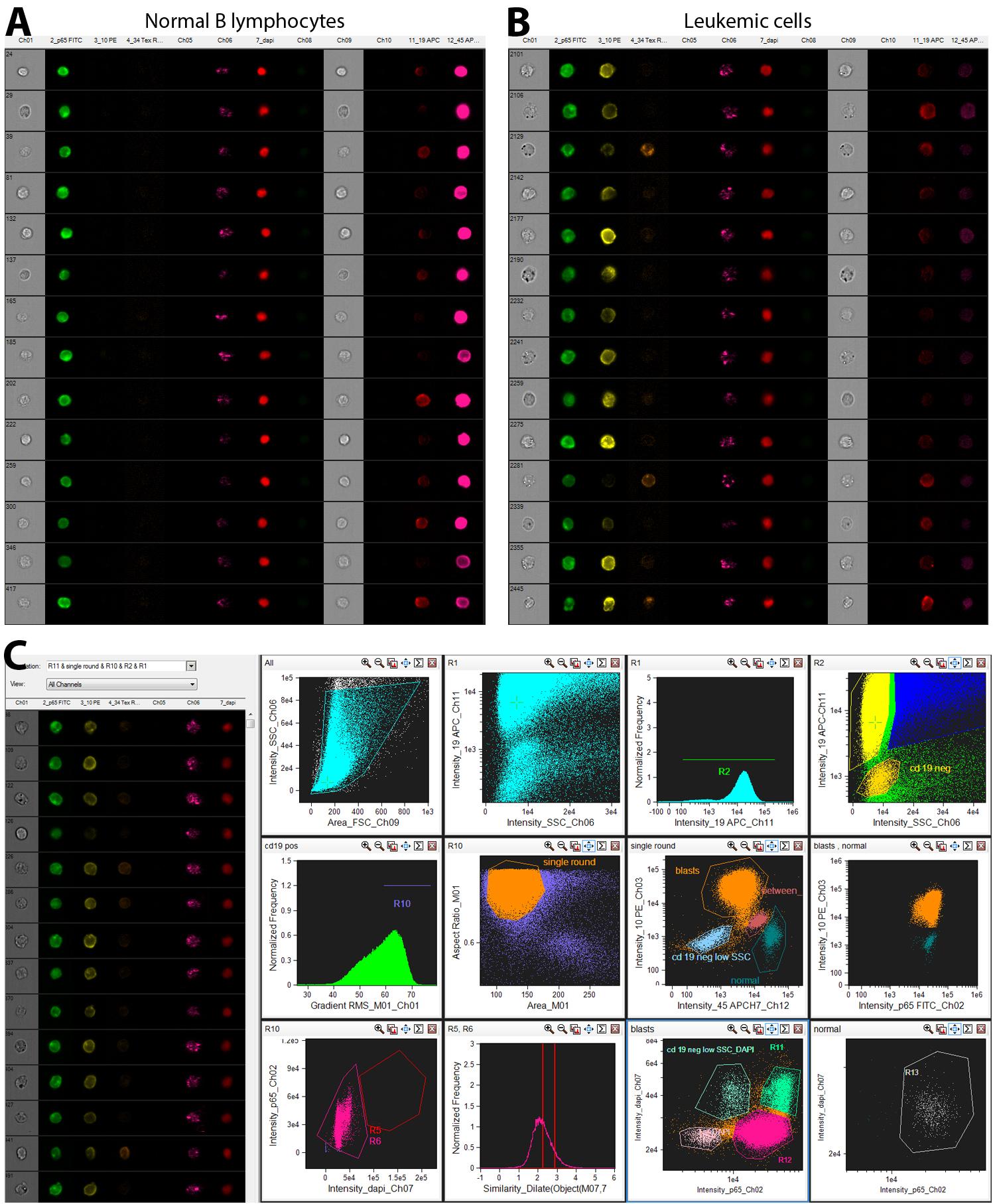

Supplement: Supplementary file 2 — Figure S1 Fluorescent biomarkers for identifying leukemic cells by imaging flow cytometry. A: Example images of normal (mature B) lymphocytes. B: Example images of lymphoblastic leukemic cells. Pseudocolors in each gallery: grayscale: bright field, green: FITC‐p65, yellow: PE‐CD10, orange: PETexas Red‐CD34, magenta: dark field, solid red: DAPI, hollow red: APC‐CD19, pink: APCH7‐CD45. C: Manual sequential gating to quantify leukemic MRD according to standard flow cytometric quantification. The gated population of normal lymphocytes (CD19+CD10—CD34— and CD45+), leukemic blasts (CD19+CD10+CD34+/— and CD45+/—) and other nucleated cells were also exported as ground‐truth training datasets for machine learning algorithms. [file CYTO-97-407-s001.tiff]

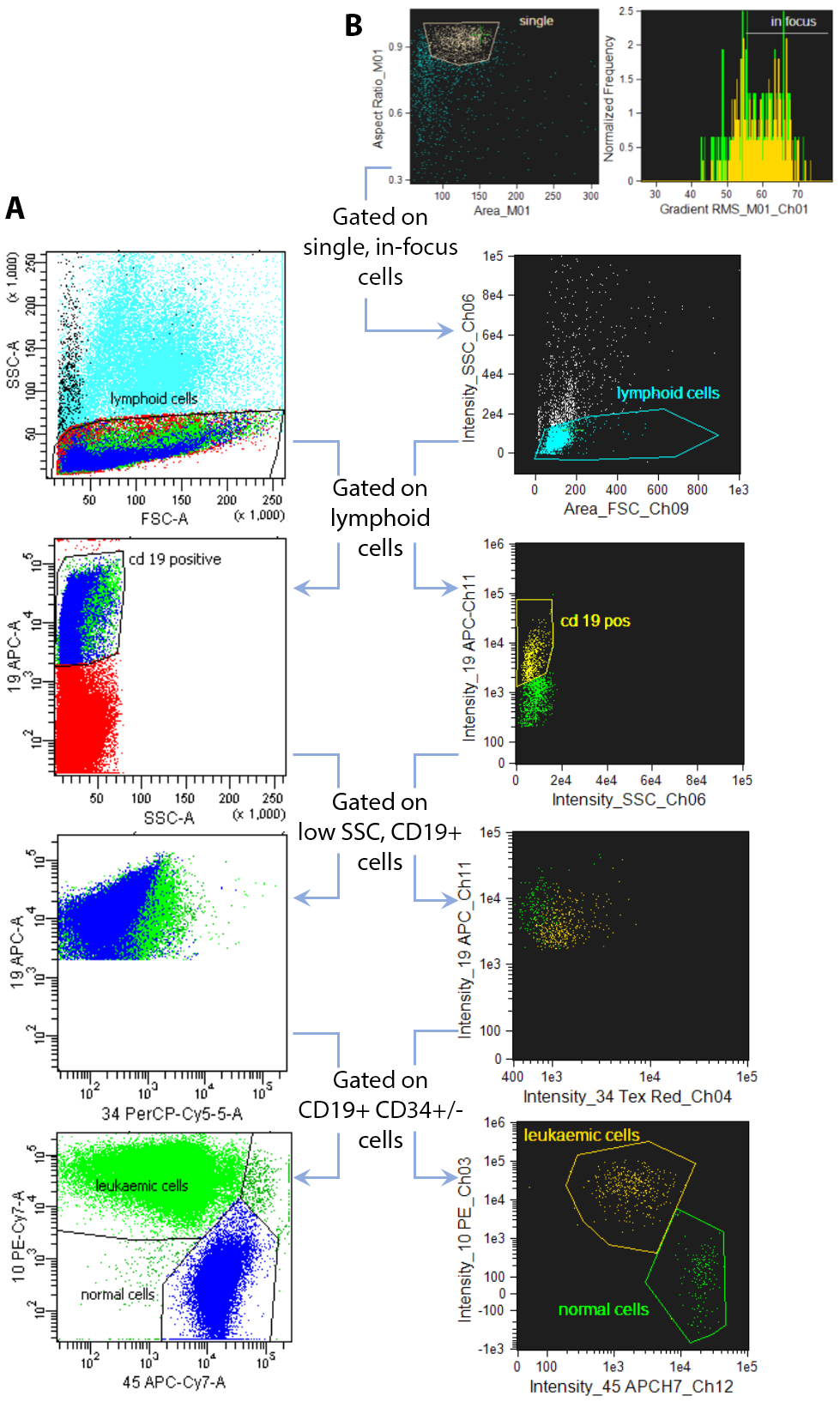

Supplement: Supplementary file 3 — Figure S2 Conventional gating template for identifying leukemia and normal B lymphocyte cell subpopulations. The conventional (and up‐to‐date, reliable) assessment of leukemic disease state was done using manual sequential gating of dot plots. A: We used DIVA software on traditional standardized flow cytometric data (without images) to discriminate leukemic blasts, normal lymphocytes, and other cell types based on fluorescently labeled antibodies (as opposed to Fig. S1C showing imaging flow cytometry gates and software). B: Equivalent manual analysis was also performed on the fluorescence channels of imaging flow cytometry data, again based on fluorescently labeled antibodies. Extra preprocessing steps (top right scatterplots) were done to exclude out‐of‐focus cells and clustered cell clumps. The multi‐step pipeline was abstracted for clarity. [file CYTO-97-407-s003.tiff]

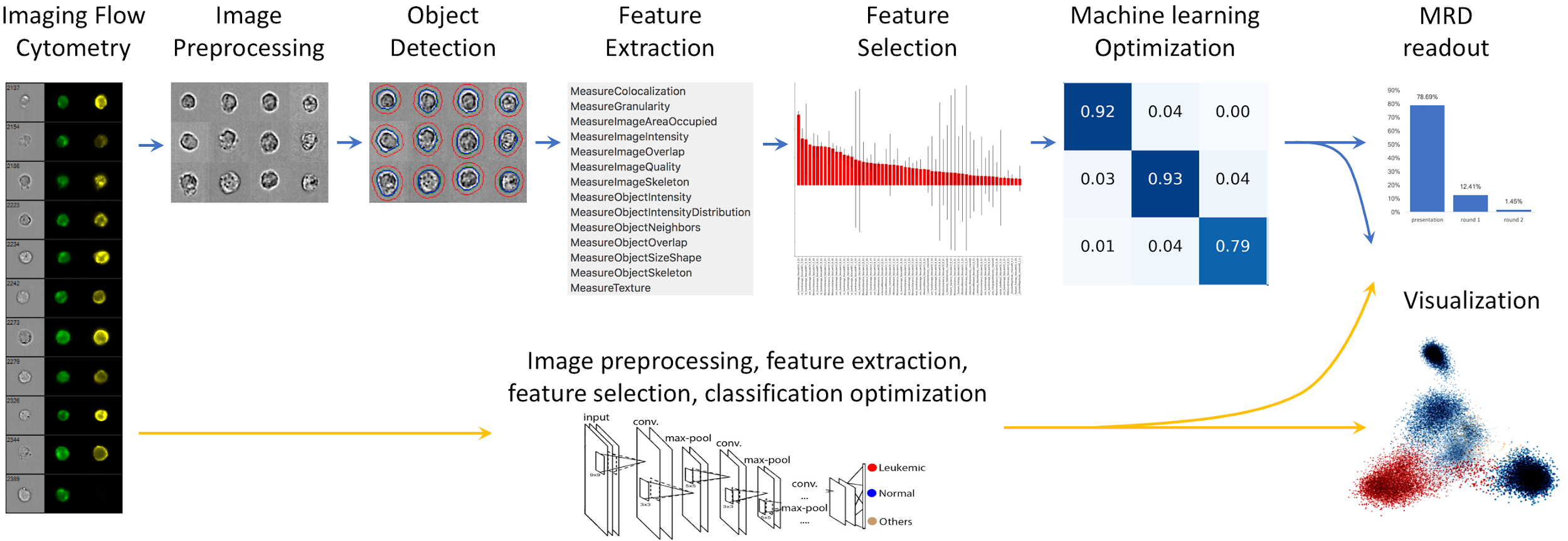

Supplement: Supplementary file 4 — Figure S3 Comparison between automated image analysis pipelines. Blue path (upper): High‐content classical image analysis protocol, which includes image preprocessing, object detection, extraction of pre‐defined morphological features by using image analysis software CellProfiler, feature selection by Cytominer, and subsequent use for traditional machine learning algorithms. Because of the sequential nature, every tuning in the upstream part of the pipeline might require complete redo of the full pipeline. Yellow path (lower): deep learning protocol, which directly takes raw images as inputs and delivers phenotypic classification as output. Using ResNet50 architecture, feature extraction, feature selection and learning optimization altogether are integrated in a single framework. The convolutional network operates with multiple levels of representation learning, in which the representation at one level (starting with the raw input images) is subsequently transformed into more abstract representations at higher levels. Higher layers of representation amplify features of the input image that are important for discrimination and suppress irrelevant variations. Classification of cells, MRD readout and data visualization are common outcomes for both protocols. [file CYTO-97-407-s004.tiff]

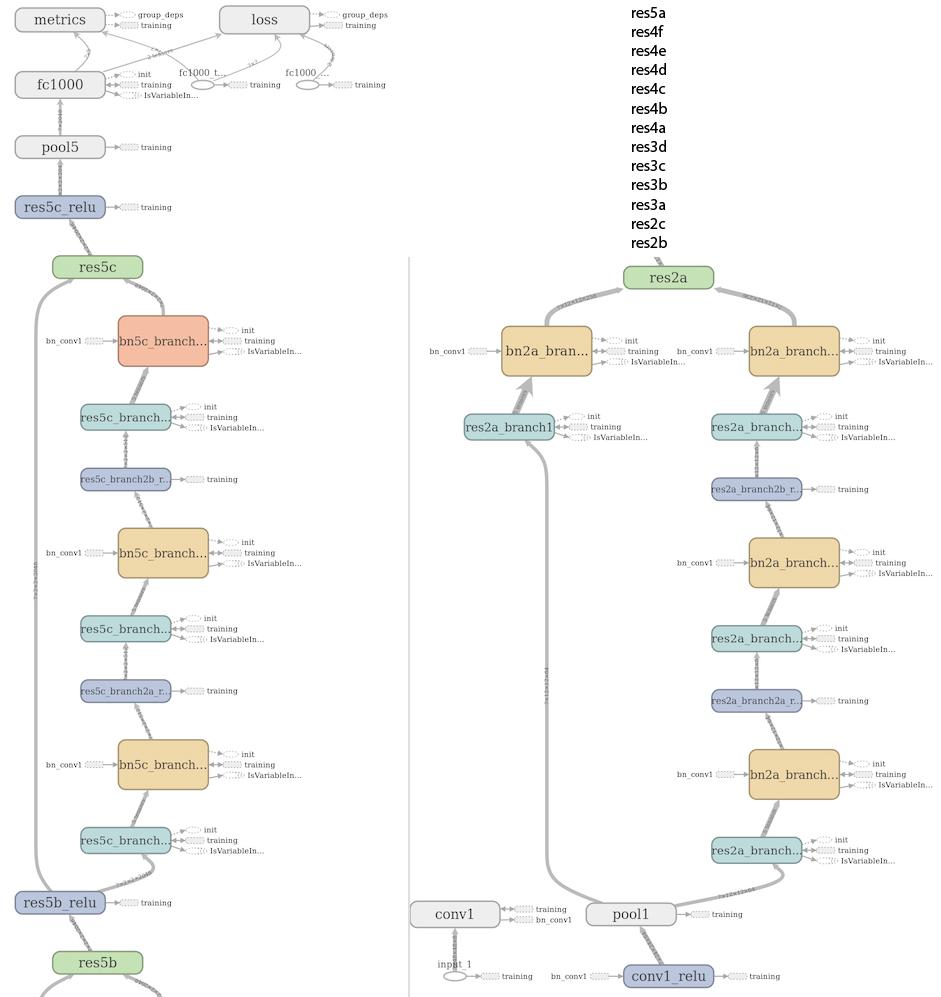

Supplement: Supplementary file 5 — Figure S4 Architecture of the neural network ResNet50 used in this study. The ResNet architecture was implemented using open‐source Tensorflow and Keras frameworks written in Python. The network includes 50 convolutional layers, forming repetitive blocks that perform residual learning, followed by fully connected and softmax layers. Bottom of left panel continues on to the top of the right panel. [file CYTO-97-407-s005.tiff]

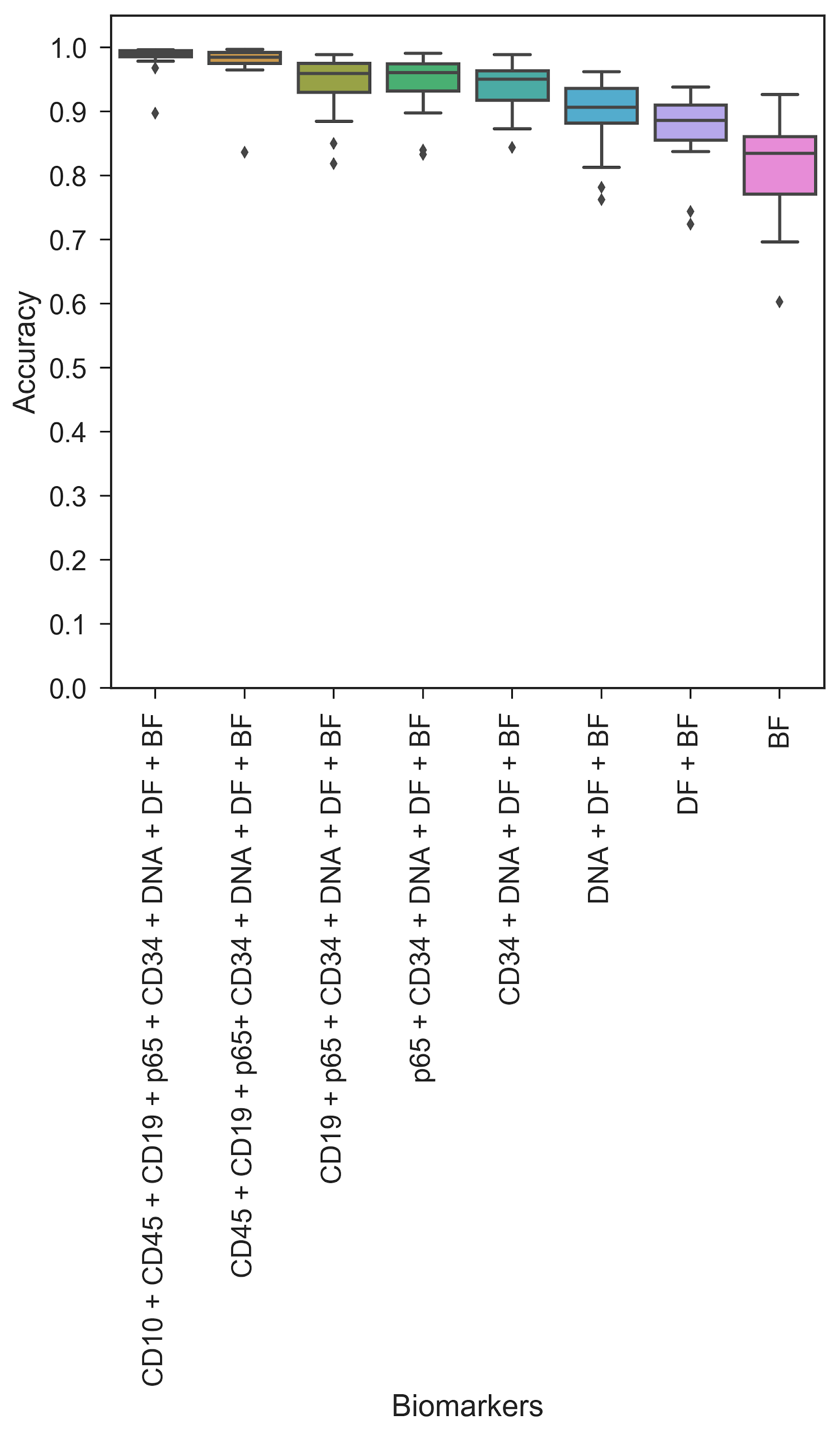

Supplement: Supplementary file 6 — Figure S5 Performance of linear support vector machine (SVM) on predicting leukemic cells, based on pre‐defined features extracted by image analysis software CellProfiler. Data from 20 patients who were at their first presentation to the clinic or after 1–4 week(s) of chemotherapy. Two cross‐validation operations were conducted in parallel: leave‐one‐label‐out (horizontal axis) and leave‐one‐patient‐out, i.e. each individual patient was tested by the classifier that was trained by a pooled dataset of 19 other patients. Boxplots show the median line, first and third quartiles. Whiskers are drawn to double interquartile range (+2IQR). Diamonds represent data events that are outside the low and high whisker ends. [file CYTO-97-407-s006.tiff]

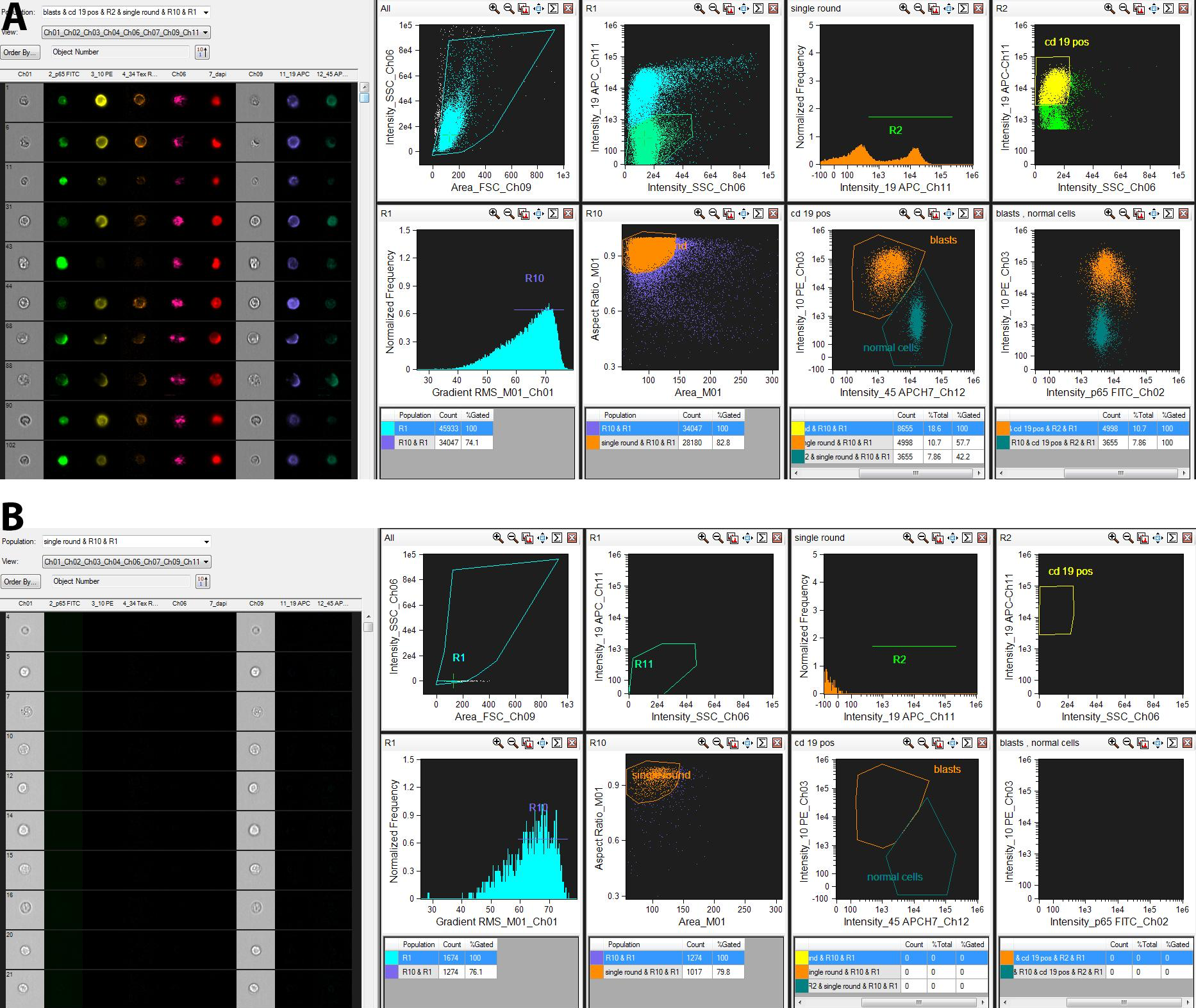

Supplement: Supplementary file 7 — Figure S6 Examination of the effects of fluorescent labeling reagents and laser excitations on the efficiency of MRD measurement by deep learning. A sample of a patient at Day 8 treatment was split into 2 portions; one was stained with fluorescent labeling reagents and one was left unstained, each was further split into two parts, one is measured with laser excitation (laser‐on) and one is with all lasers turned off. A: The fluorescently labeled sample examined in laser‐on mode. B: Unlabeled sample without laser excitation. Note that bright‐field signals (channels 1 and 9) were still visible without laser excitation and nevertheless well‐suited for deep learning algorithm. [file CYTO-97-407-s007.tiff]

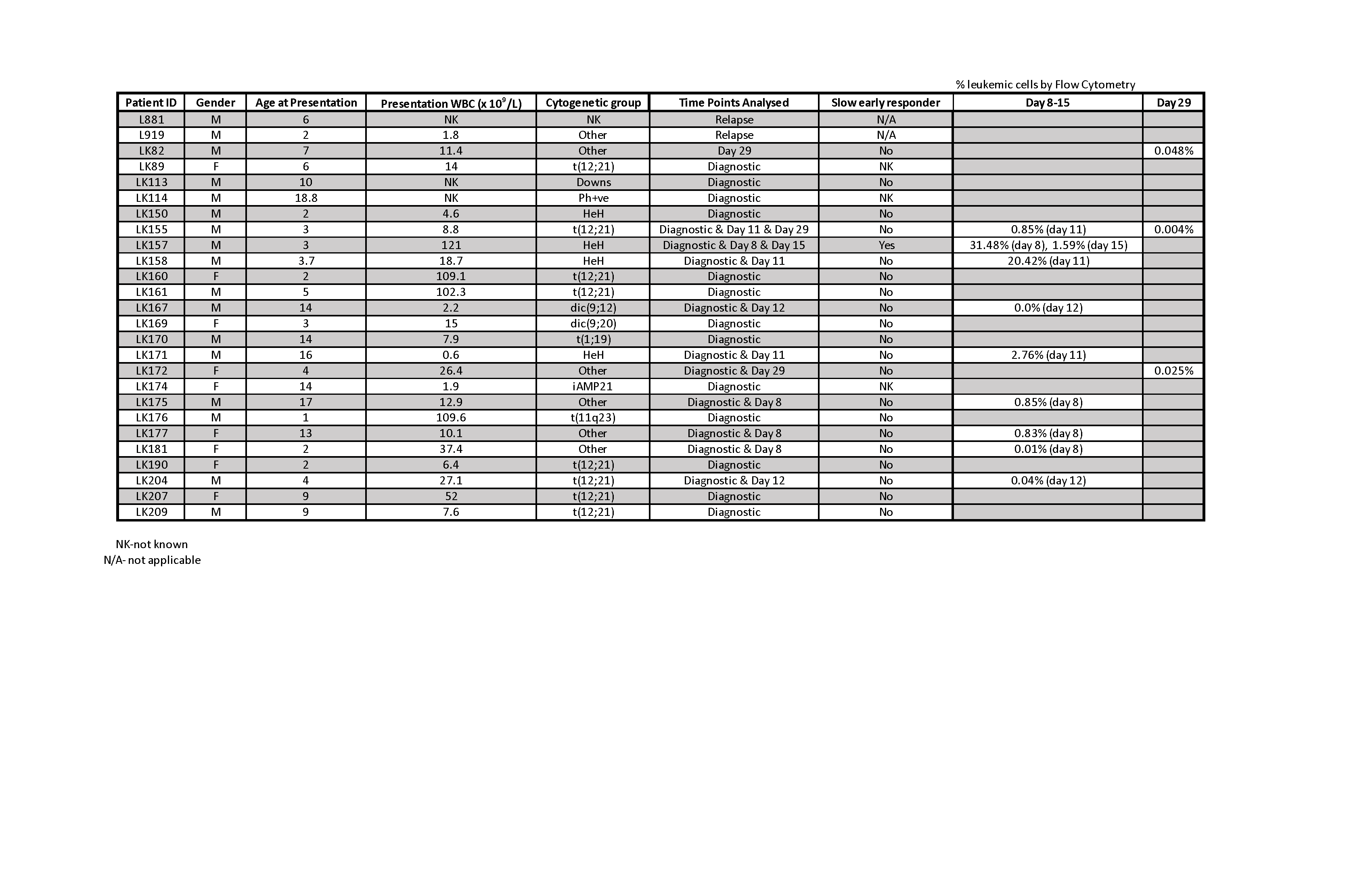

Supplement: Supplementary file 8 — Table S1 Clinical details of patients used in the study. [file CYTO-97-407-s008.tiff]
