# Supplementary material for: Label‐Free Leukemia Monitoring by Computer Vision
Source: Cytometry A. 2020 Feb 24;97(4):407–14. doi: 10.1002/cyto.a.23987 (PMC7213640; doi:10.1002/cyto.a.23987)
Supplement: Supplementary file 9 — AppendixS1: Supplementary materials [file CYTO-97-407-s009.docx]

**Supporting information: “Label-free leukemia monitoring by computer vision”, Doan *et. al.***

**Choice of Neural Network**

Before settling on the ResNet50 architecture we explored various other algorithms and networks. Here we describe our findings and comparative analysis.

In the original report of residual network, [Deep Residual Learning for Image Recognition - IEEE Conference Publication](https://ieeexplore.ieee.org/document/7780459) (2015), He et al. had compared the architectures of VGG-19 and ResNet-34, particularly in Figure 3 (partially shown below). We explored both networks and here we explain the rationale for selecting ResNet50 for our work:

**i) Computational expense**: Noticeable differences in speed can be observed throughout our experiments. Here is an attempt to explain the computing difference through the first two layers:


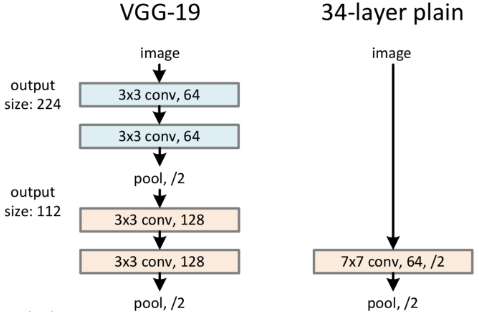


(part of Figure 3 [Deep Residual Learning for Image Recognition - IEEE Conference Publication](https://ieeexplore.ieee.org/document/7780459), 2015)

- In VGG, the first two layers apply serial convolutions on the original image frame size. In order to produce two sequential 224x224x64 outputs from the original 224x224x3 image, the first layer does ~170M floating point operations per second (FLOPs), and the next layer demands ~3.7B FLOPs.
- In contrast, ResNet first reduces the height and width of the image before convolution (denoted by “/2”), thus leads to only 240M FLOPs.

**ii) Representation learning**: For a relatively small frame size of IFC data (~48x48 mentioned in the manuscript), it is helpful for the convolutional filters to build up slowly. ResNet uses fewer kernels compared to the VGG, but it has more of them stacked alternating between convolutional operation and non-linear activation functions (the *skip connection*), which in turn helps gradient backprop not to vanish and thus enables deeper stacking of (thinner) layers:


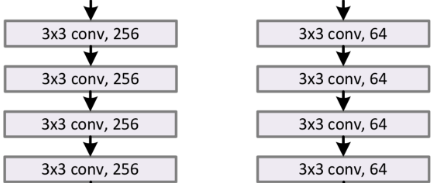


(part of Figure 3 [Deep Residual Learning for Image Recognition - IEEE Conference Publication](https://ieeexplore.ieee.org/document/7780459), 2015)

**iii) Number of (fitting) parameters**: With the explained architectures above, VGG-19 has about 143 million parameters while ResNet has 25.5 million. The number of parameters corresponds to the amount of space required to store/load the network as well as the speed of inference: for a batch size of 512, ResNet50 inference is at least 4,000 cells per second on an NVIDIA Titan X GPU, while VGG-19 is typically a few hundred cells per second. Similarly, we observed that a deeper network, such as ResNet101, did not improve the performance while being computationally expensive to train.

After preliminary trial-and-error experiments, we chose to train a newly built network over a pretrained one due to practical implementations:

- 1. Pretrained CNNs typically accept fixed size inputs: for instance, VGG16 was originally trained on an input shape of 224×224x3, Inception_V3 was trained on 299×299x3. To utilize a pre-trained network for transfer learning without significant retraining, we would need to update the input shape dimensions to accept images with different dimensions than what the original network was trained on. Doing so has significant limitations:
     1. If input image dimensions are smaller than what the CNN was trained on, there are essentially two ways to increase the input size, both negatively affecting performance:
        1. Increasing input size by upsampling: this approach introduces many artifacts, hurting loss/accuracy of the original weights, thus defeating the benefit of transfer learning (i.e. preserve the weights as much as possible).
        2. Zero (background) padding (to increase image size but keep original resolution): given the pooling (such as max-pooling) and strided convolutions, the CNN will naturally reduce volume dimensions during the forward propagation and then eventually “run out” of image features prematurely before reaching the fully connected layer.
     2. If new input images are larger than what the CNN was trained on, resizing to the original input dimensions of the pretrained CNN make small objects undetectable and in fact the original CNN may not contain enough layers to learn robust, discriminative filters for such small details. To counter such issues, we may need to tune hyperparameters exhaustively, for instance, using several learning rates, or adding additional layers to the networks. Such experiments are expensive to carry in the scope of this clinical study.
  2. The last dimension (e.g. the “x3” in 224x224x3) reflects the three channels (RGB) of everyday-life photographic images. In IFC applications, we typically have various object sizes, with multiple fluorescent channels (>3) per object, thus not suitable for the pretrained networks . Using a tweak to triple each fluorescent channel to make pseudo-RGB inputs to fit into 3-channel CNNs was indeed tested and was shown not to be cost-effective.
